# Pressure Combined with Ischemia/Reperfusion Injury Induces Deep Tissue Injury via Endoplasmic Reticulum Stress in a Rat Pressure Ulcer Model

**DOI:** 10.3390/ijms17030284

**Published:** 2016-02-25

**Authors:** Fei-Fei Cui, Ying-Ying Pan, Hao-Huang Xie, Xiao-Hui Wang, Hong-Xue Shi, Jian Xiao, Hong-Yu Zhang, Hao-Teng Chang, Li-Ping Jiang

**Affiliations:** 1 Department of Nursing, the Affiliated Dongyang People’s Hospital of Wenzhou Medical University, Jinhua 322100, China; dyhospitalcff@163.com; 2 Department of Nursing School, Wenzhou Medical University, Wenzhou 325035, China; panyingying1989@sina.com (Y.-Y.P.); xie.haohuang@163.com (H.-H.X.); 15068251331@163.com (X.-H.W.); 3 School of Pharmacy, Key Laboratory of Biotechnology and Pharmaceutical Engineering, Wenzhou Medical University, Wenzhou 325035, China; xue.henwuji@163.com (H.-X.S.); xfxj2000@126.com (J.X.); st.hyz@hotmail.com (H.-Y.Z.); 4 Graduate Institute of Basic Medical Science, China Medical University, Taichung 40402, Taiwan; 5 Department of Computer Science and Information Engineering, Asia University, Taichung 41354, Taiwan; 6 Department of Nursing, The Affiliated Xinhua Hospital of Shanghai Jiaotong University School of Medicine, Shanghai 200092, China

**Keywords:** pressure ulcer, deep tissue injury, endoplasmic reticulum stress, *Akt/GSK3β* pathway, rat model

## Abstract

Pressure ulcer is a complex and significant health problem in long-term bedridden patients, and there is currently no effective treatment or efficient prevention method. Furthermore, the molecular mechanisms and pathogenesis contributing to the deep injury of pressure ulcers are unclear. The aim of the study was to explore the role of endoplasmic reticulum (*ER*) stress and *Akt/GSK3β* signaling in pressure ulcers. A model of pressure-induced deep tissue injury in adult Sprague-Dawley rats was established. Rats were treated with 2-h compression and subsequent 0.5-h release for various cycles. After recovery, the tissue in the compressed regions was collected for further analysis. The compressed muscle tissues showed clear cellular degenerative features. First, the expression levels of *ER* stress proteins *GRP78*, *CHOP*, and *caspase-12* were generally increased compared to those in the control. Phosphorylated *Akt* and phosphorylated *GSK3β* were upregulated in the beginning of muscle compression, and immediately significantly decreased at the initiation of ischemia-reperfusion injury in compressed muscles tissue. These data show that *ER* stress may be involved in the underlying mechanisms of cell degeneration after pressure ulcers and that the *Akt/GSK3β* signal pathway may play an important role in deep tissue injury induced by pressure and ischemia/reperfusion.

## 1. Introduction

A pressure ulcer is a localized tissue injury caused by the compression of underlying tissue with bony prominence pressure [1]. The National Pressure Ulcer Advisory Panel has improved the system for evaluating the stages of a pressure ulcer and described that deep tissue injury (DTI) is characterized by damage to the underlying soft tissue such as muscles. Compared with superficial skin, subcutaneous soft tissue is an original site for pressure ulcers. This type of ulceration is severe and difficult to detect. When such an ulceration reaches an advanced stage, treatment becomes difficult and the prognosis is poor [2]. Therefore, a superficial skin focus cannot represent the entirety of pressure-induced DTI [3,4].

In addition, Gefen *et al.* found that muscle tissue is a significant site for the initiation of pressure ulcers, particularly DTI [5]. In compressed regions, skin, muscle, and connective tissues may become injured [6]. Although the mechanisms of this process have been hypothesized and intensively studied, the details regarding the molecular mechanisms remain unclear.

The process of ischemia–reperfusion (I/R) injury in pressure ulcers, which involves incomplete and indirect ischemia followed by incomplete and chronic reperfusion, differs from I/R injuries in other organs [7,8]. In recent studies, I/R injury has been regarded as the major contributing determinant in the formation of pressure ulcers [9]. Reperfusion of blood causing cellular edema, tissue damage, and overproduction of reactive oxygen species trigger a process termed oxidative stress, which may cause the accumulation of unfolded proteins in the ER lumen and disturb ER homeostasis, resulting in ER stress. ER is an organelle that may be involved in calcium homeostasis, protein folding, secretion, and lipid biosynthesis. Certain pathophysiological stimuli such as ischemia, hypoxia, I/R, and reactive oxygen species can perturb the functions of the ER and cause accumulation of unfolded proteins, resulting in ER stress [10]. 

A previous study indicated that apoptosis was triggered in the animal model of pressure-caused DTI [11]. The Akt pathway plays an important role in signal transduction pathways implicated in cell growth, apoptosis, survival, and metabolism [12]. Akt counteracts ER stress-induced cell death by suppressing caspase-12 activity and CHOP expression [13]. The serine/threonine kinase/glycogen synthase kinase 3β (Akt/GSK3β) pathway plays an important role in signal transduction pathways and has been implicated in cell growth, apoptosis, survival, and metabolism [12]. Akt activation triggers downstream protein GSK3β phosphorylation, leading to the prevention of apoptosis [14,15]; this counteracts ER stress-induced cell death [16] and suppresses the expression of cysteine aspartate-specific protease 12 (caspase-12) and C/EBP homologous protein (CHOP) activity [13]. Therefore, activation of the Akt/GSK3β pathway can be used to monitor cell fate. Since few studies have investigated the molecular mechanisms of pressure ulcers, in this study we established a rat model and focused on determining the roles of ER stress and the Akt signaling pathway in the DTI of pressure ulcers. This *in vivo* investigation examined the early-onset molecular regulation of pressure ulcers, as well as DTI induced by I/R.

## 2. Results

### 2.1. Establishment of a Pressure Ulcer Model

**Figure 1:**
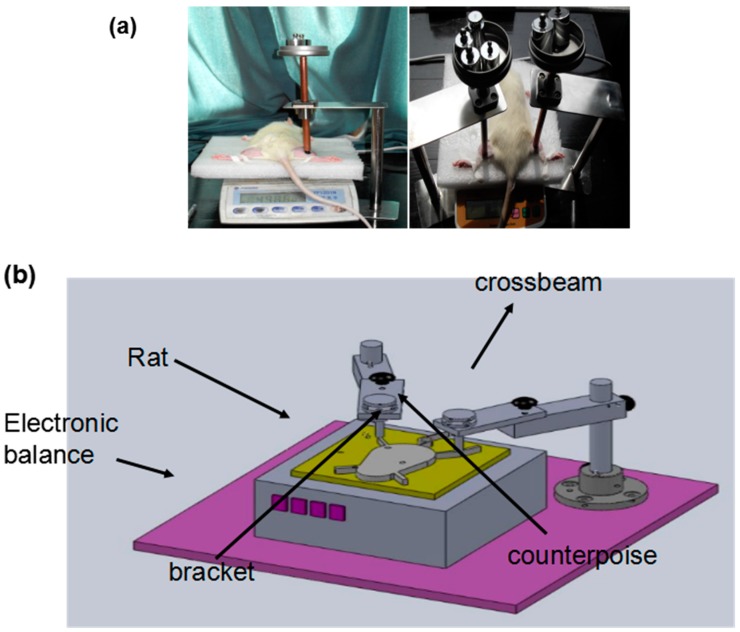
Establishment of a rat pressure ulcer model. (**a**) Application of the compression load to the shanks of a rat; (**b**) Cartoon for the device used for the rat pressure ulcer model.

In order to observe the pathology of DTI induced by pressure combined with I/R injury, rat pressure ulcers were established using the module shown in Figure 1. Briefly, 1 cm^2^ of the rat limb muscle was loaded with 230 g for 2 h and subsequently released for 0.5 h. This represents 1 cycle. This model mimicked the formative processes of I/R injury. After various cycles of compression treatment and various recovery days, the muscle tissues were isolated for pathological analyses and molecular signaling identification.

**Figure 2:**
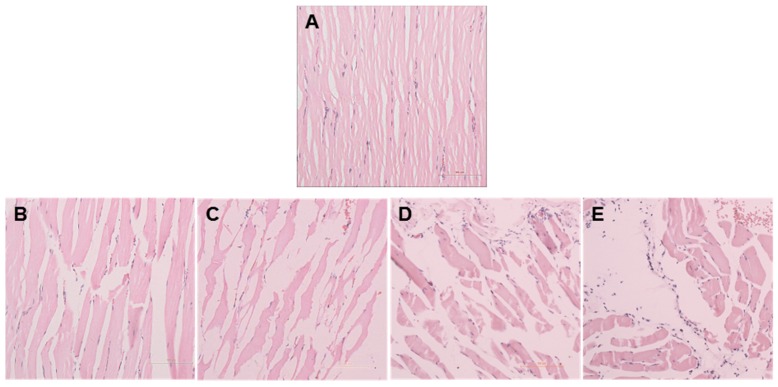

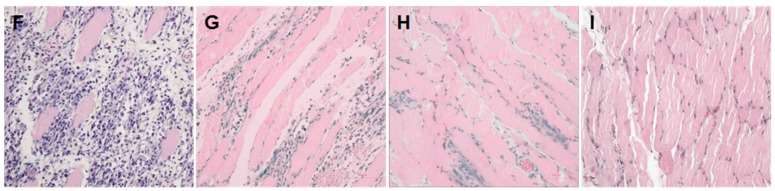
Morphology of muscle tissues. Compressed muscles were isolated at various cycles of compression and for various recovery days. Tissue slides were stained with H&E. Images were acquired using a light microscope with a ×200 objective. (**A**) (Control): The myofibers of control muscles were tightly packed; (**B**,**C**) (1 and 3 cycles): Compressed muscles tissues in the experimental group generally showed histopathological characteristics, including waxy degeneration or vacuolization; (**D**) (6 cycles): showed massive nuclei aggregation in the interstitial space; (**E**) (9 cycles): showed muscle fiber dissolution, fracture, and hyaline degeneration; (**F**) (1 day): Compressed muscles tissues in the recovery group showed multiple inflammatory cells in the necrosis area; (**G**–**I**) (3, 5, 7 days): myofiber atrophy and derangement as well as inflammatory cell infiltration were still observed.

**Figure 3:**
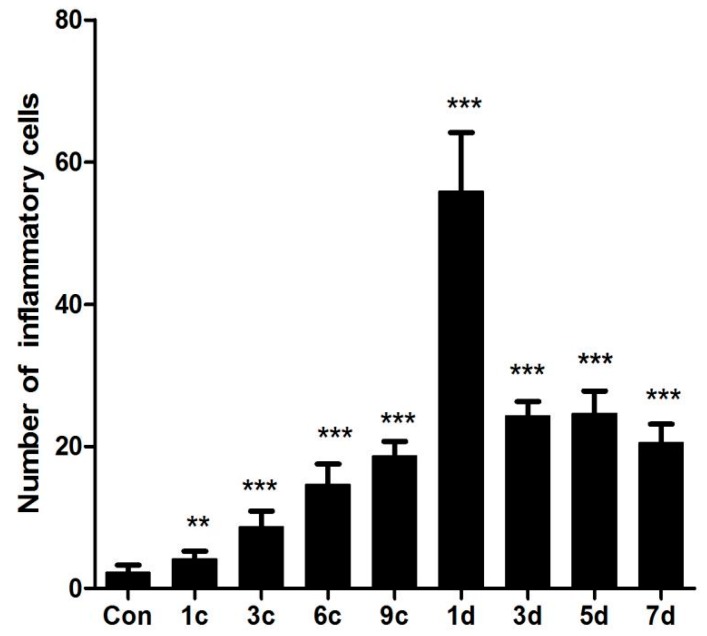
Number of inflammatory cells in compressed muscle tissue of rats in various groups (** *p* < 0.01, *** *p* < 0.001 *vs.* control group).

After the compression procedures, the muscle tissues generally showed some degenerative characteristics, including waxy degeneration or vacuolization, massive nuclei aggregation in the interstitial space, muscle fiber dissolution, and fracture and hyaline degeneration. Particularly, a region close to a bone showed apparent degenerative features. In the control group, myofibers presented angular shapes and lined up in an orderly and tight manner (Figure 2A). However, myofibers in the experimental groups were arranged loosely and massive inflammatory cells had infiltrated into the interstitial space (Figure 2B–E). In the recovery groups, myocytes were partially dissolved, while myofibers were twisted and broken. There were multiple inflammatory cells in the necrosis area (Figure 2F). The expression of inflammatory cytokines was clearly decreased; however, in the three days, five days, and seven days groups, myofiber atrophy and derangement as well as inflammatory cell infiltration were still observed (Figure 2G–I). The inflammatory cells were counted to represent the pathological scoring of the muscle damage (Figure 3).

### 2.2. ER Stress Was Observed in the Pressure Ulcer Model

**Figure 4:**
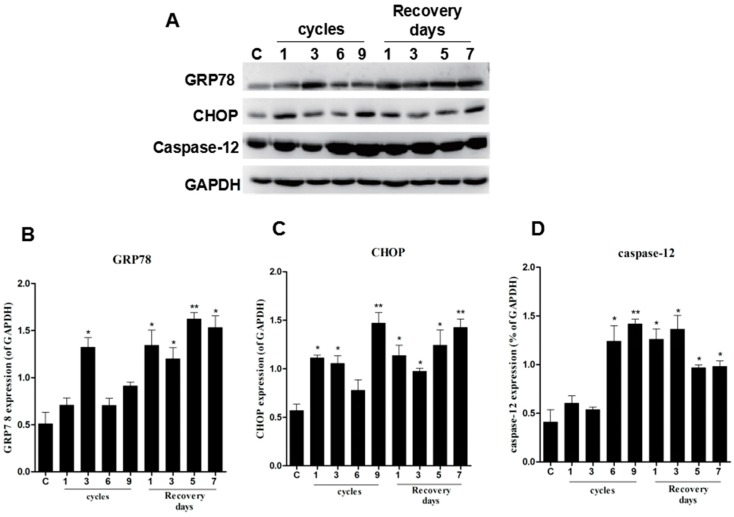
Levels of ER stress-related proteins were upregulated in compressed tissues. The protein expression of GRP78, CHOP, and cleaved caspase-12 was determined by western blotting. (**A**) GAPDH was used as a protein loading control and for band density normalization; (**B**–**D**) The optical density of GRP78, CHOP, and cleaved caspase-12 protein levels were analyzed and plotted. Data are presented as the mean values ± SD. *n* = 8. * *p* < 0.05; ** *p* < 0.01 as compared with control group.

ER stress is an important cause of cell differentiation, damage, and apoptosis [17]. In our study, glucose-regulated protein 78 (GRP78), CHOP, and caspase-12 were used to monitor the ER stress response to cell damage. After compression and I/R treatment, the onsite protein expression levels of GRP78, CHOP, and caspase-12 increased compared to the control group. GRP78 and CHOP levels were remarkably increased, showing dependence on both the compression cycles and recovery days (Figure 4A–C). Although the levels of caspase-12 increased after compression and I/R injury, caspase-12 levels were lower in the recovery group on days five and seven (Figure 4A,D). These data suggest that the cell death signaling pathway is triggered by ER stress.

**Figure 5:**
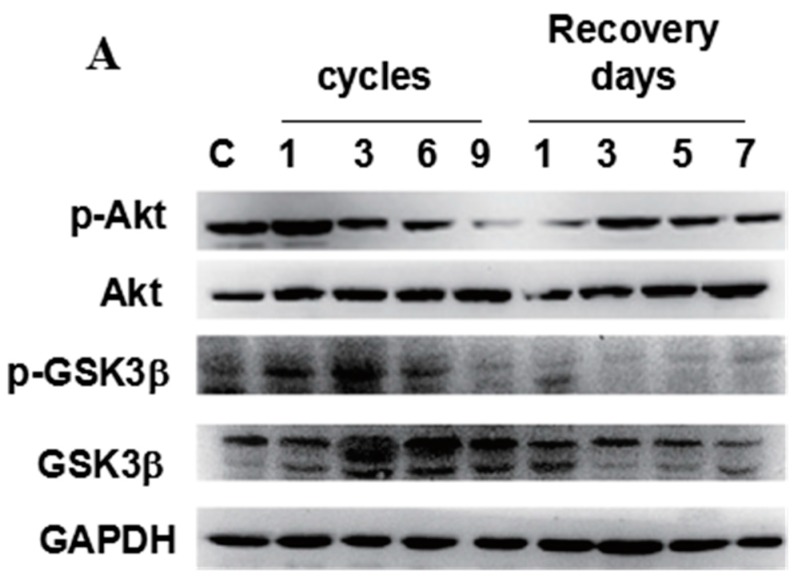

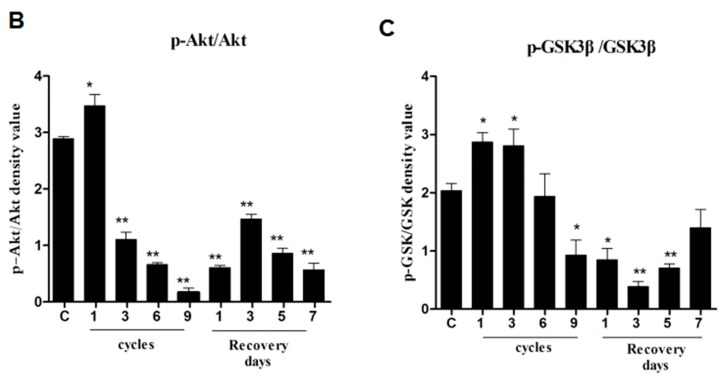
Phospho-Akt and phospho-GSK3β were downregulated in compressed tissues. The protein level of phospho-Akt (p-Akt), Akt, phospho-GSK3β (p-GSK3β), and GSK3β were determined by western blotting. (**A**) GAPDH was used as a protein loading control and for band density normalization; (**B**,**C**) Bar diagram of p-Akt/Akt and p-GSK3β/GSK3β ratios was calculated and plotted. Data are represented as the mean values ± SD. *n* = 8. * *p* < 0.05; ** *p* < 0.01 as compared with the control group (con).

In addition, Akt/GSK3β signaling is thought to be involved in the main downstream pathway of ER stress [18], in which down-regulation of phospho-Akt and phospho-GSK3β may induce cell death. The level of phospho-Akt slightly increased in the group subjected to 1 cycle of compression, but significantly decreased with increasing compression cycles and recovery days (Figure 5A,B). In contrast, the level of phospho-GSK3β initially increased and then significantly decreased during 9-cycle compression (Figure 5A,C). These results indicate that mechanical compression up-regulates the phosphorylation of both Akt and GSK3β. However, with the initiation of I/R injury, the levels of both phospho-Akt and GSK3β significantly decreased as compared to the control.

### 2.3. Correlations between CHOP, Phospho-Akt, and Phospho-GSK3β

In order to understand the relationships among CHOP, phospho-Akt, and phospho-GSK3β, their correlations were examined using Pearson’s correlation coefficient (*r*). The phospho-Akt level was found to be negatively correlated with CHOP (*r* = −0.63, *p* < 0.01) and cleaved caspase-12 (*r* = −0.72, *p* < 0.01). However, phospho-GSK3β activity was negatively correlated with caspase-12 (*r* = −0.76, *p* < 0.01) (Table 1).

## 3. Discussion

Pressure ulcers are often classified into two types, superficial and deep pressure ulcers. Although pressure ulcers have long been a problem in clinical long-term care, the molecular mechanisms of their pathogenesis remain too unclear for effective intervention and prevention [11,19,20]. In the present study, we evaluated the changes in ER stress and the Akt/GSK-3β pathway in pressure-I/R injury-induced DTI in a pressure ulcer rat model. Previous studies on pressure ulcers have concentrated on skin tissue [19]. However, muscle tissue shows lower stress tolerance and higher susceptibility to external mechanical compression [19], and thus DTI can rapidly progress to a deep pressure ulcer. Thus, from the outset, pressure ulcers likely represent a deep tissue injury, and an initial description of intact skin may not adequately reflect their true severity [20]. Therefore, the pathogenesis of muscle tissues in DTI has recently become a focus in pressure ulcer research.

Our previous research [21] showed that a mitochondria-mediated apoptotic pathway is activated in the development of early-stage PU after undergoing prolonged I/R procedures. However, the specific apoptotic pathways and their function in the pathogenesis of DTI were unclear. In the present study, we found that waxy degeneration or vacuolization, massive nuclei aggregation in interstitial space, muscle fiber dissolution, and fracture and hyaline degeneration occurred in compressed muscle tissues compared to in the uncompressed control group in a time-dependent manner. In the recovery group, inflammation was slowly alleviated when the ischemia and oxygen-poor conditions improved, but muscle fibers showed less recovery because of muscle fiber breakage and muscular necrosis. This phenomenon may be related to the sustained moderate pressure combined with I/R injury. Siu *et al.* reported that the degenerative features manifested in the bottom of the muscle tissues rather than the cutaneous tissue [11]. The results of our study are similar to those of the previous study. In addition, several studies have demonstrated the pivotal role of ER stress as a major contributor that can increase apoptosis and exacerbate cell damage after I/R. ER stress is considered an initial response of cells to stress or damage. This stress is also related to many diseases such as brain and myocardium I/R injury [22]. However, whether activation of ER stress is associated with the pathogenesis of pressure combined with I/R injury-induced DTI remains unclear.

Apoptosis has become a research hot spot in the field of tissue injury and repair, particularly in skin wound healing, chronic ulcer formation, and the field of healing [23]. ER stress-mediated apoptosis plays a key role in cell death and I/R injury [24]. Stimulation from various external sources or environments can cause an imbalance of homeostasis in the ER, resulting in the induction of ER stress. If the ER stress is strong and continuous, it can activate pro-apoptotic factors, CHOP, and caspase-12, leading to cell dysfunction and even cell death. In this study, we observed that the expression of GRP78, CHOP, and caspase-12 was increased in the experimental groups as compared to the control group. The different expression levels of these proteins may be correlated with pressure-I/R injury-induced ER homeostasis imbalance [24]. At some points during the recovery days, CHOP and caspase-12 showed inconsistent expression levels, which may have been caused by the caspase-12 independent apoptotic pathway. Zhang *et al.* also suggested that certain ER stresses protect heart functioning, and overexpression of GRP78 may protect cardiac myocytes from oxidative damage [25].

Recently, the role of mitochondria in the activation of cell death pathways has been attributed to enhanced brain damage following cerebral I/R in diabetic conditions. The release of cyt C from the mitochondria and opening of the mitochondrial permeability transition pore (MPTP) trigger ER stress caspase activation. The Akt/GSK3β pathway plays a crucial role in maintaining cell homeostasis and survival [26]. GSK3β is a multifunctional Ser/Thr kinase that plays important roles in necrosis and apoptosis, and activated GSK3β can promote cell apoptosis. It is unlikely, however, that GSK-3β is active constitutively, with phospho-GSK3β resulting in inhibiting its activity. However, Akt can prevent the apoptotic activity by phosphorylating GSK3β [27]. The inhibition of GSK3β-induced cardio protection in ischemic postconditioning (IPostC) may be related to the prevention of MPTP opening, which is a crucial event in lethal reperfusion injury.

The present study was conducted to investigate whether pressure combined with I/R injury in a pressure ulcer could inhibit the phosphorylation of Akt and the downstream target GSK3β via inhibiting Akt activity. Recent studies have supported that mitochondrial GSK3β plays an important role in myocyte necrosis after I/R injury. For instance, GSK3β inactivation (high GSK3β phosphorylation levels) has been found to alleviate MPTP opening significantly in response to I/R injury [28]. Inhibition of GSK3β-induced cardio protection in IPostC may be related to the prevention of MPTP opening. A previous study reported that significant differential pressure overload of the PI3K signaling pathway before and after induction of I/R increased in perfusion pressure during normoxia, which was associated with a significant increase in the phosphorylation of both Akt and GSK3β. However, in heart perfused at higher pressure, the initiation of I/R led to obvious decrease of phospho-Akt and phospho-GSK3β levels [29]. Our results showed that in the experimental groups, the expression of phospho-Akt was decreased and that phospho-GSK3β increased immediately in one cycle and then decreased in the experiment group during subsequent cycles. In the recovery groups, the expression of phospho-Akt reached a peak in the three days group and then gradually decreased, while phospho-GSK3β increased, reaching a peak in the seven days group. This dynamic change may be related to external mechanical compression, which up-regulates the phosphorylation of both Akt and GSK3β and the initiation of I/R injury, which significantly decreases both phospho-Akt and phospho-GSK3β levels in compressed muscle tissues. This may be because Akt inactivation is a downstream event of CHOP-mediated cell apoptosis. The inactivation of GSK-3β was triggered by Akt-induced phosphorylation, resulting in the inhibition of apoptosis. Once mechanical stress was relieved, Akt activity increased with decreasing ischemia injury, but the cell could not avoid apoptosis. However, it was unclear whether the differential expression of phospho-Akt and GSK3β was caused by a functional disorder or damage to the mitochondrial membrane in muscle tissue after pressure combined with I/R injury.

We established that abnormal activation of Akt kinase is involved in I/R injury-induced brain and myocardium damage. We observed a similar pattern in pressure combined with I/R injury in a pressure ulcer rat model. Taken together, we suggest that the molecular mechanisms of ER stress and the Akt/GSK3β signal pathway contribute to DTI of pressure ulcers. Our findings may be applied to prevent pressure ulcers in the future.

## 4. Materials and Methods

### 4.1. Animals

Male adult Sprague-Dawley rats with similar body weights of 300 ± 20 g were selected and housed in a specific pathogen-free facility at 20 °C at the Wenzhou Medical University Animal Centre. The condition of reverse light/dark cycles (12:12 h) supplemented with a standard nutrient diet and water *ad libitum* was provided to the rats during the entire study period. All experimental procedures were carried out with the approval of the Institutional Animal Ethics Subcommittee of the Wenzhou Medical University. (Approval No.: LAEC-198, Approval Date: 4 June 2013).

### 4.2. Animal Model for Deep Pressure Ulcers

Static pressure of 230 g was applied to an area of 1.0 cm^2^ in the tibial region of the limbs of the rats. The compression load was applied to each rat using a controlled indenter, where the magnitude of the compression force was continuously monitored by an electronic balance under the indenter (Figure 1). The rats were randomly divided into 4 groups referred to as 1, 3, 6, and 9 cycles, with 8 rats in each group. One cycle indicates that the rat was treated with compression for 2 h, which was then released for 0.5 h. One, 3, 6, and 9 cycles indicate that the rats were sacrificed at these cycle numbers. In the recovery groups, all rats were treated with 9 cycles of compression and released first, recovered for 1, 3, 5, and 7 days, and then sacrificed.

Uncompressed limbs served as controls. At the end of each rat’s allotted time, they were sacrificed and the compressed tissue was isolated and immediately frozen in liquid nitrogen. After 30 min, the tissue was stored at −80 °C until further analyses.

### 4.3. Histological Analysis

Hematoxylin and eosin staining was performed to evaluate the pathological condition of the tissues after compression treatment. These tissues were cut horizontally, embedded in paraffin, and sliced on a microtome with a thickness of 5 µm. The cellular degenerative histological features in tissue samples, including myofiber arrangement, angular myofiber contour, number of nuclei in the interstitial space, and myofibers, were observed in three random image fields captured using an optical microscope.

### 4.4. Western Blotting Analysis

A 30-µg protein extract was re-suspended in SDS-PAGE loading buffer, boiled at 95 °C for 10 min, separated by 11.5% SDS-PAGE, and then transferred onto a polyvinylidene difluoride membrane. After protein transfer, the membranes were incubated with Tris-buffered saline containing 0.1% Tween 20 (TBST) with 5% non-fat milk for 1 h blocking at room temperature with shaking, and with the corresponding primary antibodies, anti-GAPDH (1:1000, Santa Cruz Biotech, Paso Robles, CA, USA), anti-GRP78 (1:300, Santa Cruz Biotech, Paso Robles, CA, USA), anti-CHOP (1:300, Santa Cruz Biotech), anti-Akt and anti-p-Akt (1:300, Santa Cruz Biotech), anti-GSK3β and anti-p-GSK3β (1:300, Santa Cruz Biotech), and anti-caspase-12 (1:1000, Abcam, Cambridge, UK) at 4 °C overnight.

After incubation in TBS, the membranes were washed three times using TBST, and subsequently incubated with horseradish peroxidase-conjugated secondary antibodies (1:3000, Santa Cruz Biotech) at room temperature for 2 h. The signals were visualized using the ChemiDocTM XRS+ Imaging System, and band densities were quantified using Multi Gauge Software of Science Lab 2006 (FUJIFILM Corporation, Tokyo, Japan).

### 4.5. Statistical Analysis

Data were expressed as the mean ± SD. Statistical analyses were carried out using SPSS 17.0 (SPSS, Inc., Chicago, IL, USA). One-way ANOVA was used to compare differences between the experimental groups and the control group. An LSD-*t* test was used to compare multiple groups. Pearson correlation was performed to determine the correlation among the CHOP, phosphor-Akt, and phosphor-GSK3β. Statistical significance was accepted at *p* < 0.05.

## Figures and Tables

**Table 1:** Correlations between CHOP, phospho-Akt, and phospho-GSK3β.

Correlated Factors	*r* ^a^	*p*
p-Akt and CHOP	−0.63	< 0.01
p-Akt and cleaved caspase-12	−0.72	< 0.01
p-GSK3β and CHOP	−0.28	> 0.05
p-GSK3β cleaved caspase-12	−0.76	< 0.01

^a^ Correlation was analyzed using Pearson’s correlation.

## Abbreviations

DTI: Deep tissue injury
Akt/GSK3β: Serine/threonine Kinase /Glycogen synthase kinase 3β
ER: Endoplasmic reticulum
CHOP: C/EBP homologous protein
IPostC: Ischemic postconditioning
I/R: Ischemia/reperfusion
MPTP: Mitochondrial permeability transition pores
GRP78: Glucose regulated protein 78
Caspase-12: Cysteine aspartate-specific protease 12
